# Two-Photon Absorption Response of Functionalized BODIPY Dyes in Near-IR Region by Tuning Conjugation Length and Meso-Substituents

**DOI:** 10.1021/acsomega.3c02314

**Published:** 2023-08-16

**Authors:** Elif Akhuseyin Yildiz, Bekir Asilcan Ünlü, Ahmet Karatay, Yasemin Bozkurt, Muhammed Emre Özler, Fazlı Sözmen, Ebru Yabaş, Bahadir Boyacioglu, Hüseyin Ünver, Ayhan Elmali

**Affiliations:** † Department of Physics Engineering, Faculty of Engineering,Ankara University, 06100 Beşevler-Ankara, Türkiye; ‡ Department of Metallurgical and Materials Engineering,Sivas Cumhuriyet University, 58140Sivas, Türkiye; § Nanotechnology Engineering Department, Faculty of Engineering,Sivas Cumhuriyet University, 58140 Sivas, Türkiye; ∥ Advanced Technology Application and Research Center,Sivas Cumhuriyet University, 58140 Sivas, Türkiye; ⊥ Vocational School of Health Services,Ankara University, 06290 Kecioren-Ankara, Türkiye; # Department of Physics, Faculty of Science,Ankara University, 06100 Besevler-Ankara, Türkiye

## Abstract

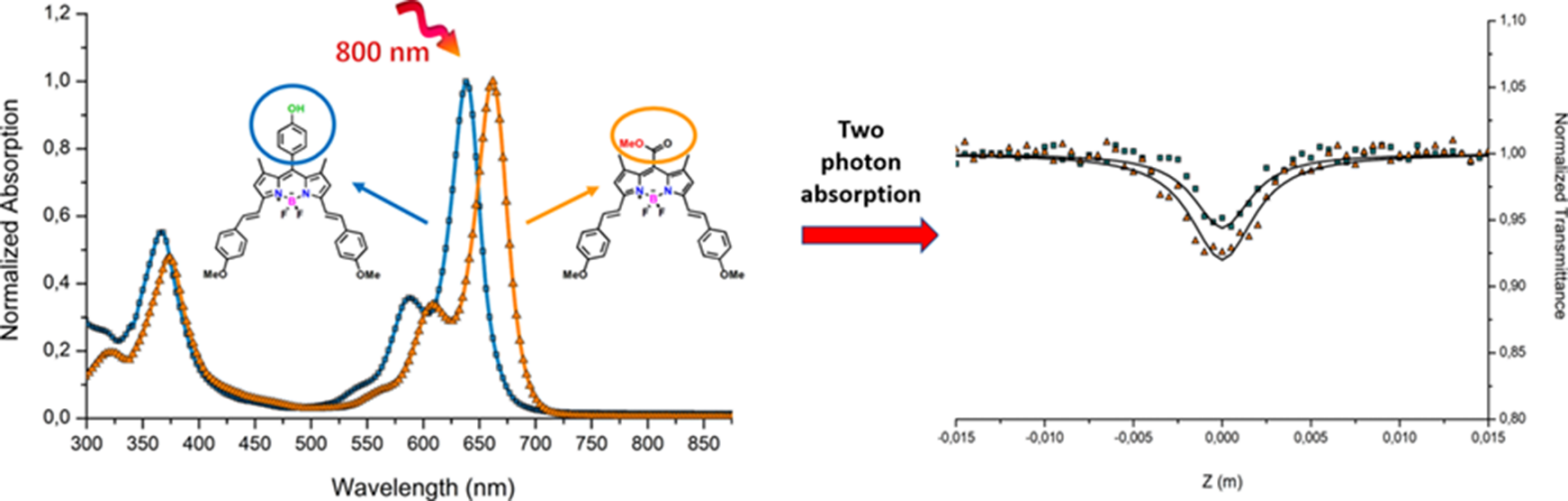

BODIPY dyes substituted by phenol or −COOMe units at the meso-position (C8) with and without a distyryl group including a methoxy moiety at the -C3 and -C5 positions of the BODIPY have been synthesized to analyze the photophysical properties. To clarify the ground-state interaction, absorption and emission features were investigated in the THF environment. Extending the π-conjugation with the methoxy moiety at -C3 and -C5 positions of BODIPY leads to a spectral shifting of the absorption maxima toward red by 120 nm. In addition, attaching the −COOMe unit at the meso-position of the BODIPY structure increases nonradiative molecular relaxation as compared to compounds possessing phenol substituents at the same position. We have investigated the effect of phenol and a −COOMe group and π-extended conjugation length with a methoxy moiety on the properties of two-photon absorption (TPA) and electron transfer dynamics by performing open-aperture (OA) Z-scan and femtosecond transient absorption spectroscopy measurements, respectively. The synthesized BODIPY compounds with the distyryl group including the methoxy unit show TPA character due to the longer conjugation length and therefore intramolecular charge transfer ability. Based on the OA Z-scan experiments upon photoexcitation with 800 nm pulsed laser light, TPA cross-section values were obtained as 74 and 81 GM for the compounds possessing phenol and −COOMe units at the meso-position of BODIPY treated by distyryl group with methoxy moieties, respectively. Additionally, optical and electronic properties were calculated theoretically by using the DFT method.

## 1 Introduction

The chromophores exhibiting two-photon absorption (TPA) character are of particular attention in a diversified amount of potential applications, which include optical information storage,^1,2^ optical limiting devices designed to protect sensors from laser damages,^3^ two-photon fluorescence and imaging microscopy for bioimaging,^4,5^ and photodynamic therapy applied for cancer treatment.^6,7^ One of the most important challenges for the improvement of TPA cross-section (TPCS) value is to design and synthesize new organic chromophores.^8,9^ Designing novel organic molecules that exhibit high TPCS values within the wavelength range of 700–1100 nm holds particular significance for phototheranostic applications such as biological imaging and photodynamic therapy.^10^ The functionalization of the molecular structures by substituting efficient donor–acceptor groups and heavy atoms and extending π-conjugated chains enables the enhancement of TPCS values.^11−13^ However, it is desired to design halogens and transition-metal-free organic molecules which play an important role in improving and progressing biomedical sciences. Therefore, molecular engineering plays a crucial role in designing and synthesizing novel heavy-atom-free organic compounds for bioimaging and photodynamic therapy applications.

4,4-Difluoro-4-bora-3a,4a-diaza-s-indacene (BODIPY) chromophores are miscellaneous chromophores that have attracted extensive attention in recent decades. The BODIPY chromophores exhibit excellent photophysical properties, including a large molar extinction coefficient in the visible and near-IR regions, high fluorescence quantum yield, photochemical and relatively high thermal stability, and high TPA responses.^8,9,14,15^ The photophysical characteristics of the BODIPY dyes can be readily altered by chemical modifications. For instance, the wavelength corresponding to the absorption band in the linear absorption spectra of the BODIPY chromophores can be adjusted finely to the near-IR region (∼650 nm) by the reaction of Knoevenagel condensation. This significant spectral change enables absorption and/or emission in longer wavelength regions and, therefore, greater tissue penetration for photodynamic therapy. As a result, the BODIPY dyes possessing a broad absorption band in the near-IR area are in high demand for using long wavelength range applications. In this regard, much effort has been expended in developing sophisticated styryl-BODIPYs to extend the absorption band and construct the frontier orbital levels of BODIPYs.^16^ Especially, the attachment of the styryl group with the electron-donating or withdrawing moieties in -C3,-C5 or -C1,-C7 positions of BODIPY presents particular interest. For this purpose, introducing the electron donor and/or acceptor groups with an extension of π-conjugation length leads to increasing the charge transfer ability and therefore improves the TPA property as well as the TPCS value.

The present work is focused on the synthesis and photophysical characterization of novel BODIPY chromophores possessing phenol or −COOMe units at the meso-position with and without a distyryl group including a methoxy unit. Steady-state absorption properties as well as emission features of the studied compounds were fully analyzed in detail. To further investigate the effect of phenol or −COOMe substitutes and π-expanded conjugation length with a methoxy unit on charge transfer mechanisms, femtosecond transient absorption spectroscopy experiments were conducted in a THF environment. Although there have been a few studies on the synthesis and characterization of phenol or −COOMe substituted at the meso-position of BODIPY, investigations on the TPA properties of these compounds are lacking in the literature.^17,18^ Therefore, to present the relation between the molecular structure and two-photon absorption properties, an open-air Z-scan technique was conducted. Furthermore, theoretical calculation studies based upon density functional theory (DFT) were performed in addition to the experimental studies.

## 2 Materials and Methods

### 2.1 Materials

The reactions utilized reagents and solvents of reagent-grade quality. Flash column chromatography (FCC) was carried out using Merck Silica gel 60 with particle sizes ranging from 0.040 to 0.063 mm and 230–400 mesh ASTM. The reactions were monitored through thin layer chromatography (TLC) employing silica gel plates (Merck Silica Gel PF-254). This procedure was repeated for all of the reactions in the study.

### 2.2 Synthesis of Compounds

#### 2.2.1 Synthesis of Compound **1**

The synthesis of compound **1**, as described in refs (19) and (20) is outlined below.

First, 4-hydroxybenzaldehyde (0.122 g, 1 mmol) and 2,4-dimethyl-pyrrole (0.209 g, 2.2 mmol) were dissolved in 90 mL of THF. Several drops of trifluoroacetic acid were added to this solution, and the mixture was stirred at room temperature for 10 h. Next, a solution of DDQ (0.227 g, 1 mmol) in 120 mL of THF was introduced to the medium, and the reaction mixture was stirred for an additional 4 h.

Subsequently, 9 mL of triethylamine (0.08 mol) was added to the mixture and stirred for 15 min. After that, BF_3_·OEt_2_ (9 mL, 0.08 mol) was slowly added dropwise to the cooled mixture in an ice–water bath. The resulting mixture was stirred overnight, and then the reaction solvent was removed under reduced pressure.

The residue obtained was dissolved in 100 mL of CH_2_Cl_2_, and the organic phase was sequentially washed with 100 mL of 5% aqueous NaHCO_3_ and 2 × 100 mL of water. The washed organic phase was dried with anhydrous Na_2_SO_4_, and the solvent was removed under reduced pressure.

The product was further purified using flash silica column chromatography with a 1:1 mixture of ethyl acetate and hexane (v/v), yielding a 30% product yield. The ^1^H NMR spectrum (400 MHz, CDCl_3_) showed signals at δ_H_ 7.12 (2H, d, *J* = 8 Hz), 6.94 (2H, d, *J* = 8 Hz), 5.96 (2H, s), 2.53 (6H, s), and 1.42 (6H, s). The high-resolution mass spectrometry (HRMS) (TOF-ESI) analysis revealed a calculated *m*/*z* value of 339.1480 and an observed *m*/*z* value of 339.1468 [M – H]^−^, with a difference of 3.5 ppm.

#### 2.2.2 Synthesis of Compound **2**

Compound **2** was synthesized by the Knoevenagel condensation reaction.^20,21^ Initially, a solution of compound **1** (29.35 mg, 0.862 mmol) and *p*-methoxy benzaldehyde (46.97 mg, 0.345 mmol) was prepared in 50 mL of benzene. Then, 0.3 mL of piperidine and 0.2 mL of acetic acid were added to this solution, and the resulting mixture was heated under reflux using a Dean–Stark trap. The progress of the reaction was monitored by TLC (using a 1:1 mixture of ethyl acetate and hexane, v/v).

Once the reaction was complete, the mixture was cooled to room temperature, and the solvent was removed. 100 mL of water was added to the residue, and the product was extracted into chloroform (3 × 100 mL). The organic phase was dried using Na_2_SO_4_, and after the evaporation of the organic solvent, the product was purified using flash silica column chromatography with a 1:1 mixture of ethyl acetate and hexane (v/v), resulting in a 44% yield.

The ^1^H NMR spectrum (400 MHz, CDCl_3_) showed signals at δ_H_ 7.61 (2H, d, *J* = 16 Hz), 7.58 (4H, d, *J* = 8 Hz), 7.27–7.21 (4H, m), 7.15 (2H, d, *J* = 8 Hz), 6.96 (2H, d, *J* = 12 Hz), 6.93 (4H, d, *J* = 8 Hz), 6.59 (2H, s), 3.84 (6H, s), and 1.49 (6H, s). The high-resolution mass spectrometry (HRMS) analysis (TOF-ESI) showed a calculated *m*/*z* value of 576.2396 and an observed *m*/*z* value of 576.2291 [M]^+^, with a difference of 18.22 ppm.

#### 2.2.3 Synthesis of Compound **3**

Compound **3** was synthesized following the procedure described in ref (22). Initially, 10 mL of dry DCM was combined with 2,4-dimethyl pyrrole (8.21 mmol, 850 μL) and cooled to −78 °C. A solution of methyl-chlorooxalate (3.265 mmol, 0.3 mL) in 4 mL of dry DCM was then added dropwise to the reaction flask. The reaction mixture was stirred at −78 °C for 4 h under an argon atmosphere.

Subsequently, triethylamine (13.06 mmol, 1.82 mL) and BF_3_·OEt_2_ (2.60 mL) were added to the reaction, and the mixture was stirred at room temperature for an additional 2 h. The progress of the reaction was monitored by TLC (using a 3:1 mixture of hexane and ethyl acetate, v/v). The organic solvent was removed under reduced pressure. The resulting compound 3 was purified using flash silica column chromatography with a 3:1 mixture of hexane and ethyl acetate (v/v), yielding a 61% product.

The ^1^H NMR spectrum (400 MHz, CDCl3) displayed signals at δ_H_ 6.04 (2H, s), 3.94 (3H, s), 2.50 (6H, s), and 2.09 (6H, s). The high-resolution mass spectrometry (HRMS) analysis (TOF-ESI) revealed a calculated *m*/*z* value of 306.1351 and an observed *m*/*z* value of 306.8218 [M]^+^, with a difference of 2243.1 ppm.

#### 2.2.4 Synthesis of Compound **4**

Compound **4** was synthesized using the Knoevenagel condensation reaction, as described in refs (23) and (24). To a solution of compound **3** (22.87 mg, 0.0747 mmol) and *p*-methoxy benzaldehyde (22.377 mg, 0.14 mmol) in 50 mL of benzene, piperidine (0.3 mL) and a small amount of acetic acid (AcOH) were added. The reaction mixture was refluxed with a Dean–Stark trap, and the progress of the reaction was monitored by TLC (using a 1:1 mixture of dichloromethane and hexane, v/v).

After the completion of the reaction, the mixture was cooled to room temperature, and the solvent was evaporated. 100 mL of water was added to the residue, and the product was extracted into chloroform (3 × 100 mL). The organic phase was dried with Na_2_SO_4_, and the solvent was evaporated. The product was further purified using flash silica column chromatography with a 1:1 mixture of dichloromethane and hexane (v/v), resulting in a 44% yield.

The ^1^H NMR spectrum (400 MHz, CDCl_3_) showed signals at δ_H_ 7.58–7.55 (4H, m), 7.52 (2H, d, *J* = 16 Hz), 7.21 (2H, d, *J* = 16 Hz), 6.93–6.90 (4H, m), 6.68 (2H, s), 3.97 (3H, s), 3.84 (6H, s), and 2.16 (6H, s). The high-resolution mass spectrometry (HRMS) analysis (TOF-ESI) displayed a calculated *m*/*z* value of 543.3882 and an observed *m*/*z* value of 543.4704 [M + H]^+^, with a difference of 151.27 ppm.

### 2.3 Optical Measurements

The absorption spectra were obtained with a scanning spectrophotometer (Shimadzu UV-1800). To monitor the emission features of the studied compounds, a fluorescence spectrometer (PerkinElmer model LS 55) was used. The linear absorption and emission features of the compounds were investigated in 1 × 1 cm quartz cuvettes in a THF solution environment.

Femtosecond transient absorption spectroscopy measurements were conducted to analyze the charge transfer dynamics and decay kinetics. A commercial ultrafast pump–probe spectroscopy system provided by Helios was used. The pump and the probe pulses were generated by Ti:sapphire laser regenerative amplifier and optical parametric amplifier (OPA) systems that have 1 kHz repetition rate and 52 fs pulse duration. The wavelength of the pump was determined by the wavelength of maximum absorbance in the linear absorption spectra, while the white light continuum is the probe light. The experimental data were investigated utilizing the Surface Xplorer software program provided by Ultrafast System.

In an attempt to reveal the two-photon absorption properties of the BODIPY compounds, an open-angle Z-scan technique was used. A mode-locked Ti:Sapphire laser system provided femtosecond pulses with 800 nm pump wavelength, 1 kHz repetition rate, and 1 ps pulse duration. For two-photon absorption measurements, the solution concentration is adjusted to 5 × 10^–3^ M in 1 mm cuvette length, and 800 nm pulsed laser beam is focused on the sample by a lens with a focal length of 20 cm.

### 2.4 Computational Studies

A DFT analysis with the B3LYP/Lanl2dz basis set in the ground state^25−27^ of investigated compounds was conducted in the Gaussian 09W software package^28^ by optimizing the possible geometries obtained from the 5.0 visualization program.^29^ We used the time-dependent density functional theory (TD-DFT)/CAM-B3LYP method with a density Gauss double-ζ with the polarization function (DGDZVP) basis set^30^ in THF solvent to calculate the UV–vis spectra, frontier molecular orbital (FMO) energies, and molecular electrostatic potential (MEP) surfaces to compare with the experimental results. This study revealed that the outcomes obtained from this functional closely match the experimental results concerning the optical and electronic properties. Additionally, we computed the interfragment charge transfer (IFCT) of these compounds using Multiwfn software.^31^

## 3 Results and Discussion

### 3.1 Steady-State Absorption and Fluorescence Measurements

**Figure 1:**
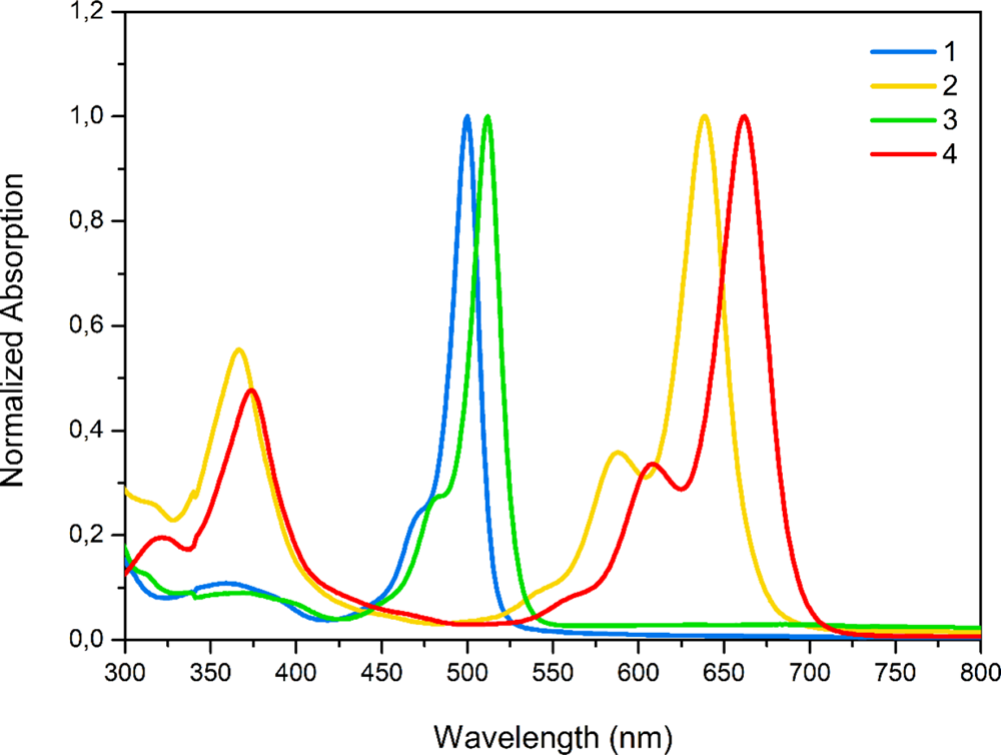
UV–vis absorption spectra of compounds **1**–**4** in THF solution, *c* = 5 × 10^–5^ M.

**Scheme 1:**
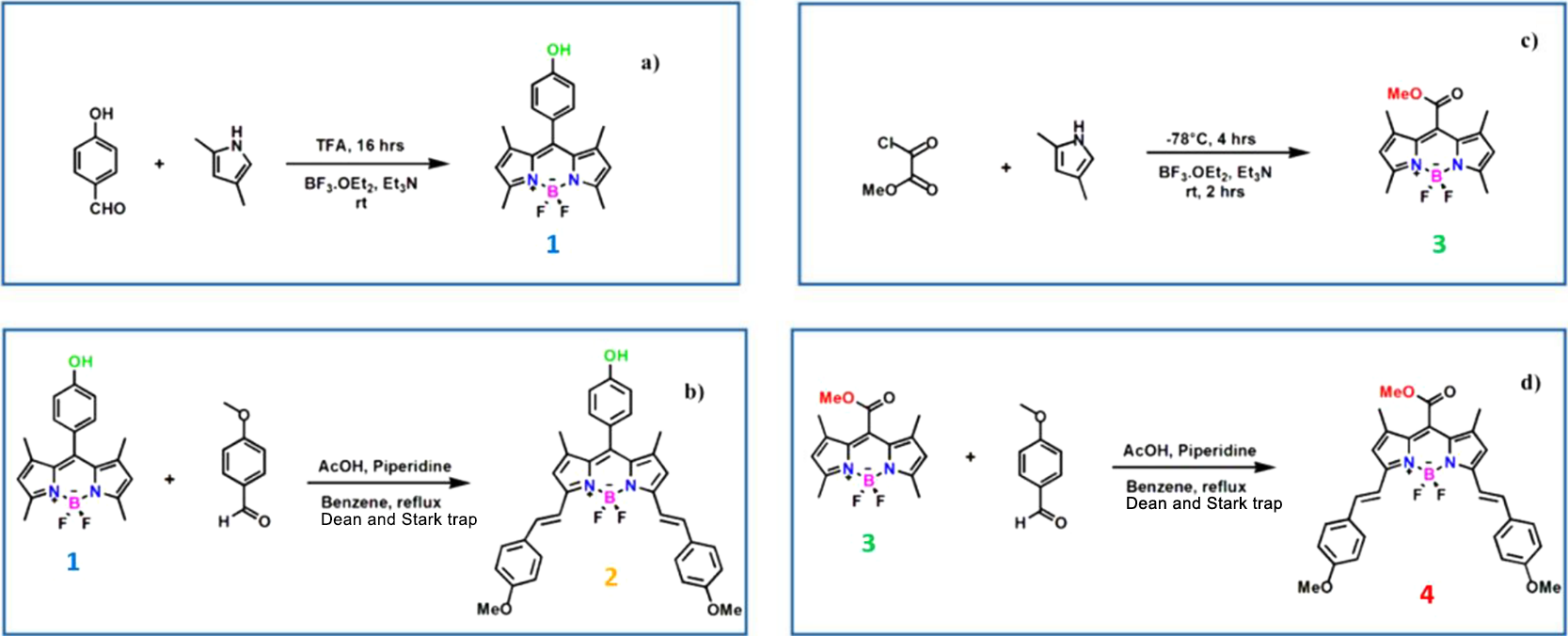
Schematic Illustration of Syntheses of BODIPY Dyes: (a) Compound **1**, (b) Compound **2**, (c) Compound **3,** and (d) Compound **4**

The BODIPY compounds with a phenol or −COOMe unit, incorporated with and without a distyryl group, including the methoxy unit at the -C3 and -C5 positions of the BODIPY core and their syntheses are schematically presented in Scheme 1. Compounds **1** and **3** were obtained from the routine BODIPY reaction of the corresponding aldehyde compounds with 2,4-dimethyl pyrrole, while compounds **2** and **4** were obtained from the Knoevenagel condensation reaction of these compounds with *p*-methoxy benzaldehyde, respectively. The linear absorption spectra of compounds **1**–**4** are indicated in Figure 1. The meso-substituted compounds **1** and **3** show the main absorption band at 500 and 512 nm, with the typical 0–1 vibrational band as a shoulder around 475 and 480 nm, respectively. The intense absorption band observed around 500 nm corresponding to the low-energy S_0_ → S_1_ transition is the signature of absorption of the BODIPY chromophore. In contrast to the phenol moiety, the −COOMe substituent leads to a 12 nm red shift of this characteristic BODIPY peak.

Extending the conjugation length at the -C3 and -C5 positions of BODIPY is a well-known method for red-shifting the absorption and emission maxima. The introduction of the distyryl group including the methoxy unit at the -C3 and -C5 positions extends the π-system delocalization and leads to a bathochromic shift from 500 and 512 nm to 639 and 653 nm for compounds **2** and **4**, respectively, as compared to the meso-substituted compounds **1** and **3**. The absorption bands which are observed as a shoulder around 588 and 610 nm for compounds **2** and **4**, respectively, can be attributed to the S_0_ → S_1_ transition with the typical vibrational band. The observed absorption bands with a weaker intensity in the blue region around 370 nm are attributed to S_0_ → S_2_ transitions for the whole studied compounds.

**Figure 2:**
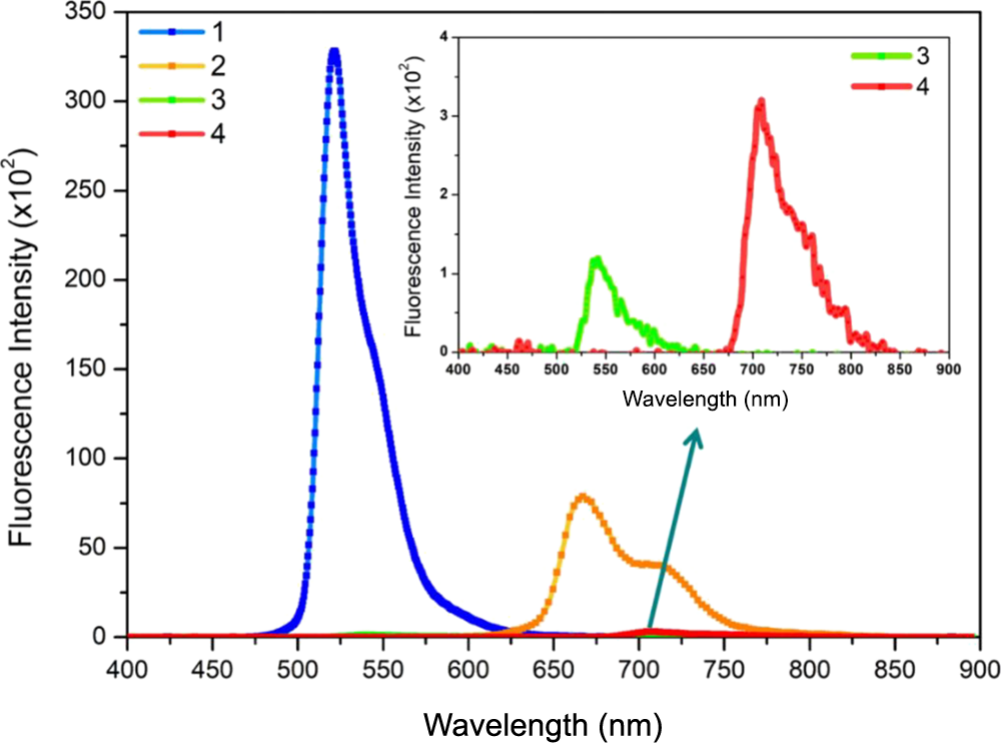
Fluorescence spectra of compounds **1**–**4** in a THF solution. The inset shows the absorption and fluorescence spectra of compounds **1** and **2** in THF solution.

Figure 2 depicts the emission spectra of BODIPY dyes of 5 × 10^–5^ M concentration in THF. The figure demonstrates that compounds **1** and **2** possessing a phenol unit at the meso-position demonstrate strong fluorescence signals. Following photoexcitation at 500 and 639 nm wavelengths, the maximum fluorescence signal intensity is located at 521 nm for compound **1** and 667 nm for compound **2**. As seen in the fluorescence spectra, the fluorescence intensity of compound **2** is only 25% of that of compound **1**. That is, the fluorescence intensity was quenched by 75% with the attachment of the distyryl group, including the methoxy unit at the α-positions on BODIPY. On the other hand, it was observed that the compounds incorporating the −COOMe group at the meso-position of the BODIPY core demonstrate nonradiative decay. We propose that the reduction of the fluorescence signal may be attributed to the excited-state lifetime due to the photoinduced electron transfer process. The presence of the −COOMe group causes an increment of the charge transfer character and a decrement in the fluorescence intensity of the compound. The fluorescence quantum yields were obtained for all the studied compounds utilizing the fluorescence data of rhodamine B in a DCM environment. The fluorescence quantum yields were found to be 0.24, 0.07, 0.001, and 0.009 for **1**, **2**, **3,** and **4**, respectively. To get deep insights into and clarify the fluorescence quenching mechanisms as well as charge transfer dynamics, time- and wavelength-dependent ultrafast pump–probe spectroscopy experiments were carried out.

### 3.2 Femtosecond Transient Absorption Spectroscopy Studies

**Figure 3:**
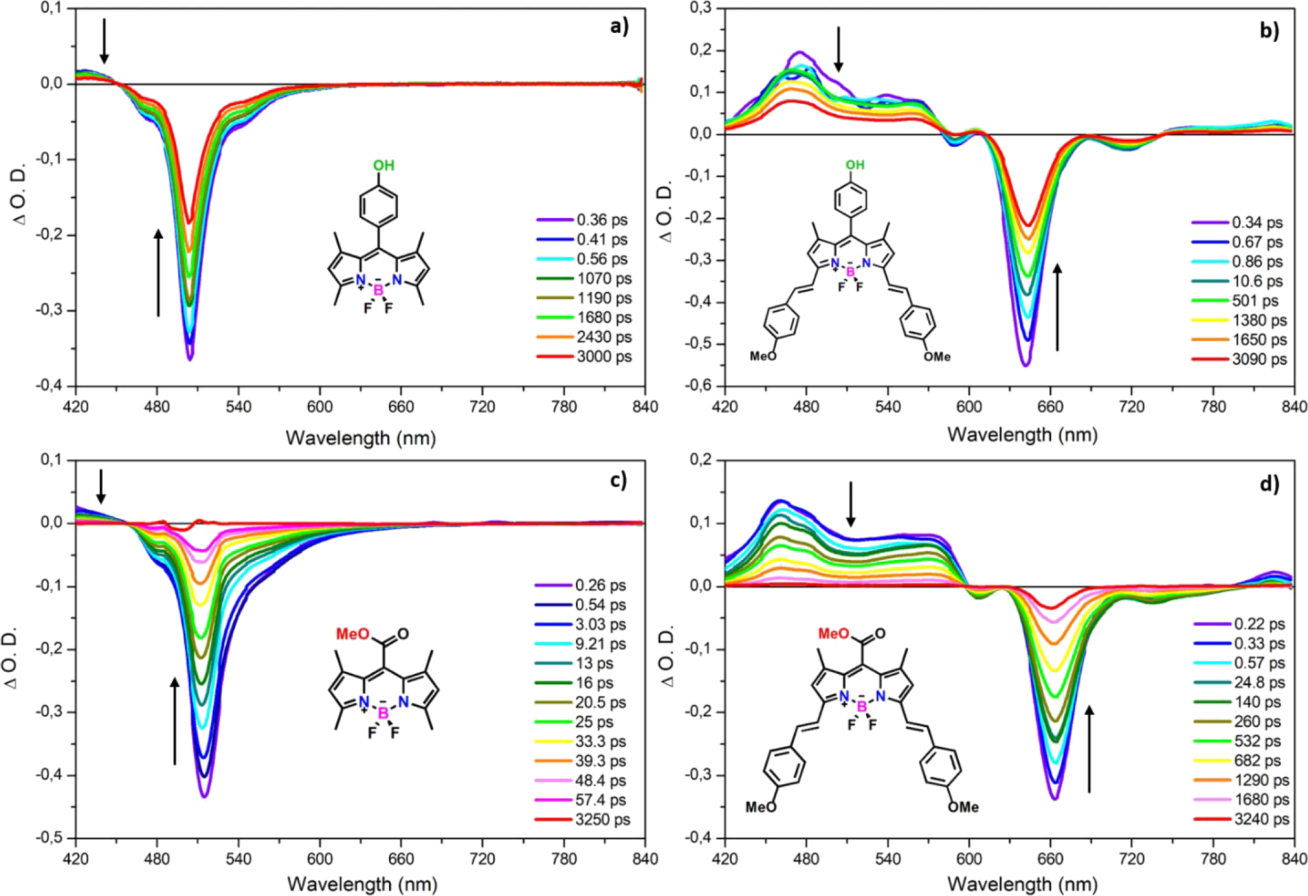
Transient absorption spectra of compounds (a) **1**, (b) **2,** (c) **3,** and (d) **4** in THF with different time delays.

To study ultrafast excited-state dynamics, charge separation, and decay kinetics, femtosecond transient absorption spectroscopy measurements were performed on the BODIPY compounds **1**–**4** in a THF environment. The transient absorption spectra of compounds **1** and **3** upon pulsed laser excitation at 500 nm are shown in Figure 3a,c. As shown in the figures, compounds **1** and **3** demonstrate similar transient absorption characteristic behaviors in the ultrafast pump–probe spectral data, with minor differences. A negative signal at 510 nm that corresponds to ground-state bleaching (GSB) and a tail that can be attributed to the stimulated emission (SE) in the range of 525 and 600 nm represent the reflection of the emission signal, as seen in the femtosecond transient absorption spectra of compounds **1** and **3**. As demonstrated in Figure 3a,c, the bleaching signal around 510 nm decreases from the zero-time delay up to 3 ns. Additionally, in the ultrafast pump–probe spectra of compounds **1** and **3**, positive signals located above 460 nm are ascribed to excited-state absorption (ESA). The absorption signal around 460 nm occurs simultaneously with the pump pulse and can be ascribed to the S_1_ → S_*n*_ transition. Likewise, compounds **2** and **4** exhibit similar transient absorption spectroscopic behaviors, as indicated in Figure 3b,d. In the transient absorption spectra of compound **2**, the GSB signals at 640 nm represent the depletion of the ground state, which is in accordance with the main absorption band in the linear absorption spectra. Besides, the weaker negative signal around 590 nm competing with the ESA signal around 607 nm corresponds to the shoulder of the main absorption band in the linear absorption spectra. Moreover, there is an extra negative signal lying around 715 nm, which can be attributed to SE, which is the reflection of the emission and charge transfer state (CTS) for compound **2**. On the other hand, the GSB band is localized around 660 nm, with the tail lying around 735 nm, corresponding to SE and CTS in the transient absorption spectra of compound **4**. Similarly, there is an additional negative signal at 607 nm matching the linear absorption spectra. In addition to all these spectral observations, there is a broad ESA signal localized below around 590 nm and above around 760 nm, corresponding to S_1_–S_*n*_ transitions for compounds **2** and **4**. In order to prove that there is a charge transfer state, we draw the decay kinetics of the GSB signal and charge transfer state (CTS) by the related wavelength, as seen in Figure S9. As seen in the inset of the figure, when the GSB signal decreases, the CTS signal increases at initial time delays. It means that the electrons located at the singlet excited state transfer to CTS in the order of a few hundred femtosecond time range. The decay rates of ESA and GSB are also compared for compounds **2** and **4** in THF. Both ESA and GSB profiles have the same decay rate, since they are all singlet-state signals, as seen in Figure S10. On the other hand, in order to examine the solvent effect on the charge transfer process clearly, we also performed ultrafast pump–probe spectroscopy experiments with different polarities, in toluene and acetonitrile (ACN) environments. As seen in Figure S11, the plotted decay kinetics of the GSB signal vary depending on the solvent polarity. This discrepancy originated from the localization of the charge transfer state. As it is well known from the literature, the energy difference between the singlet excited state and CTS is closer in nonpolar solvents. Therefore, the decay kinetics of GSB is slower in CAN as compared to that in toluene.

**Figure 4:**
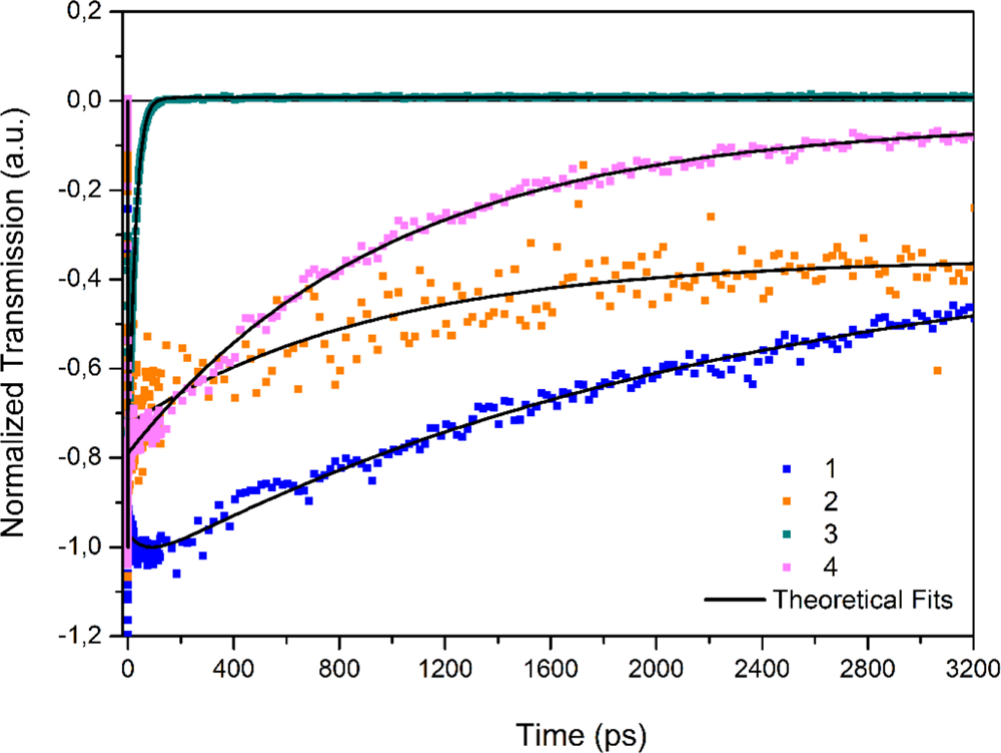
Time evolution of the bleach signals for compounds **1**–**4** in THF.

In an attempt to determine excited-state lifetimes, the decay kinetics of the studied compounds were monitored and fitted using a multiexponential fitting function at their bleach wavelength, as indicated in Figure 4 and the time components are given in Table 1. Upon pulsed laser excitation, the bleaching signal of the BODIPY compounds with the phenol unit at the meso-position (compounds **1** and **2**) exhibits a slow decaying process, which is in accordance with their fluorescence character. Compound **2** has a shorter excited-state lifetime as compared to compound **1** as it possesses a π-expanded conjugation length with a methoxy unit and leads to an intramolecular charge transfer process. On the other hand, it was monitored that the excited-state lifetime of compounds **3** and **4** decay with both fast and slow time components by probing 515 and 665 nm, respectively. The observed fast development of the bleaching signal is owed to the prompt excitation of the −COOMe unit at the meso-positions, while the slow time component is ascribed to charge recombination upon femtosecond laser excitation (Figure 4). Although compound **4** possesses a π-expanded conjugation length, it was observed that compound **4** has a longer time component as compared to compound **3**. This unexpected result may be attributed to the direct binding of the carbonyl unit of −COOMe to BODIPY, slightly extending the electron delocalization of compound **3**. This can facilitate the movements of electrons, making them more readily available for PET and ICT. Compound **4** was synthesized from compound **3** with *p*-methoxy benzaldehyde by the Knoevenagel condensation reaction. Thus, compound **4** shifted to a longer absorption wavelength with ICT, and the free electrons on the methoxy group also participate in electron delocalization. By changing the electronic structures and energy levels of the donor and acceptor in the molecule, ICT can affect the rate and efficiency of PET.

**Table 1:** Time Components Obtained from Decay Kinetics for Compounds **1**–**4** in THF

compound	τ_1_ (ps)	τ_2_ (ps)	τ_3_ (ps)
**1**	70 ± 8.7	2324 ± 162	infinity
**2**	0.5 ± 0.03	1027 ± 114	infinity
**3**	27 ± 1.62		
**4**	0.4 ± 0.06	1016 ± 102	infinity

### 3.3 OA Z-Scan Experiments

**Figure 5:**
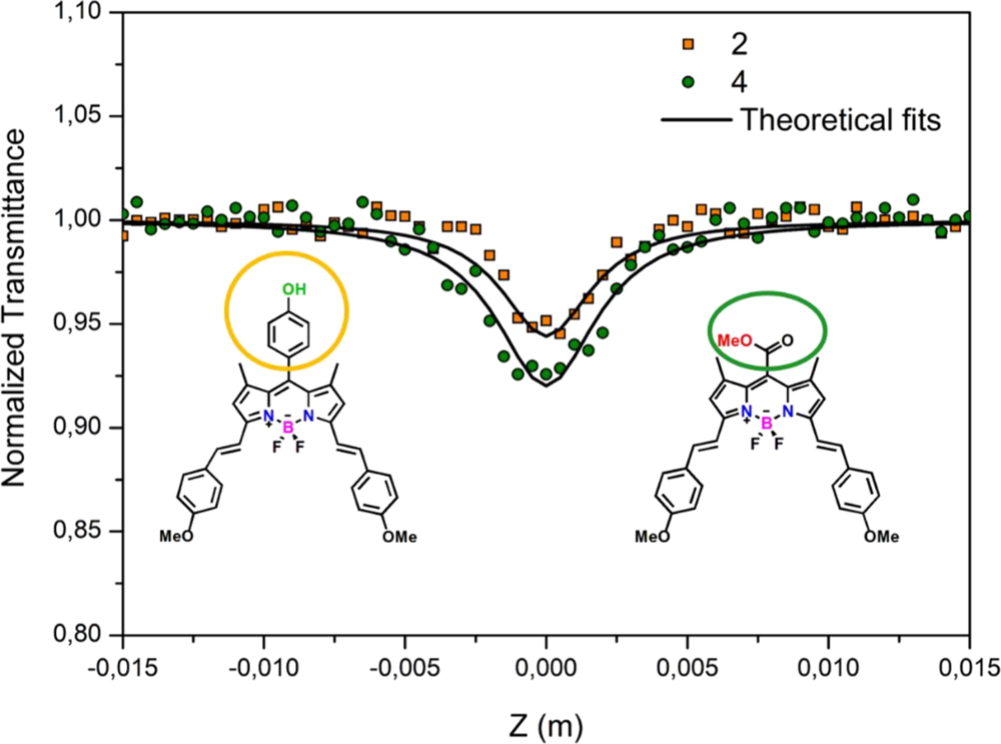
OA Z-scan experimental results (symbols) and theoretical fits (solid lines) for compounds **2** and **4** at 80 GW cm^–2^ input intensity (800 nm fs pulses) in THF.

The z-scan technique is a convenient method for detecting intensity-dependent transmission and can be utilized to measure the TPA cross-section (TPCS) value. According to the performed femtosecond transient absorption spectroscopy measurements, compounds **2** and **4** have intramolecular charge transfer property owing to the π-expanded conjugation length of the distyryl group including the methoxy unit. The intramolecular charge transfer behavior may also demonstrate that the compounds have a TPA character. Thus, OA Z-scan experiments were performed at 800 nm for compounds **2** and **4** in THF solution. To calculate the TPA coefficient (β), the nonlinear transmittance *T* depending on the laser intensity *I*_0_ is given by the following equation:
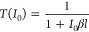
1where *l* is the optical path length. In an attempt to determine a two-photon absorption cross-section value σ_2_ (1 GM = 10^–50^ cm^4^ s photon ^–1^), the β value is obtained by fitting the OA Z-scan experimental data, and then the beta value is used in the following equation:
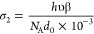
2where *N*_A_ is the Avogadro number, and *d*_0_ denotes the molar concentration of the solution. Figure 5 depicts the OA Z-scan experimental results and theoretical fits of the studied compounds at 80 GW cm^–2^ peak intensities with an 800 nm wavelength. According to the linear absorption spectra, the absorption band can be shifted toward the near-IR region, and the nonlinear optical properties can be improved with the attachment of the electron-donating groups. In the molecular structures of compounds **2** and **4**, the methoxy groups are considered electron-donating substituents and lead to a bathochromic shift, as indicated in Figure 1. In addition, if the electron-donating groups attach to the π-conjugated group at the -C3 and -C5 positions of BODIPY, the TPA properties enhance due to the intramolecular charge transfer. Consequently, the TPCS values increase by the methoxy unit possessing electron-donating nature and π-expanded conjugation length of the distyryl group improving the charge transfer mechanism. According to the fitting results, the TPCS values were achieved as 74 and 81 GM for compound **2** and compound **4**, respectively. Because of the fact that the charge transfer rates have an effect on the TPA features, compound **4** has a greater TPCS value due to the fast development of the excited state than that of compound **2**. These TPCS values are lower than the values with triphenylamine moieties at different positions of BODIPY (452, 688, and 220 GM) in our studies previously reported.^8,9,32^ On the other hand, compounds **1** and **3** which have substituents only at the meso-position of the BODIPY core did not exhibit any TPA properties at 800 nm wavelength as expected, since these moieties at the meso-position seem to not affect the TPA features.

### 3.4 UV–vis, FMO, MEP Surface, and IFCT Analyses

In this part of the research, we focused on analyzing the UV–vis spectra, the electronic structure, and behavior of the studied compounds in relation to FMO energy levels, which play an important role in various electronic processes and reactivity, and on the study of MEP surfaces, which provide valuable information about the distribution of electron density and electrostatic properties of molecules. To achieve this, we used the TDDFT method, a powerful computational approach widely used to study the electronic properties of molecules. Furthermore, besides FMO energies and MEP analysis, we performed theoretical calculations for compounds **1**–**4** to determine their IFCT properties, which is another important way to gain insights into the electronic structure, energy transfer, and reactivity of complex molecular systems, helping to understand various chemical and biological processes at the molecular level. In the THF environment, the UV–vis spectra of the compounds studied were theoretically analyzed, and Table 2 gives details of the spectroscopic characteristics of the electronic transitions, including absorption wavelengths, excitation energies, oscillator strengths, and major contributions. For compound **1**, we found maximum absorbance values at 473 nm (HOMO → LUMO with 98% contribution) and 331 nm (H-2 → LUMO with 96% contribution) at excitation energies of 2.62 and 3.75 eV, respectively. Compound **2** displays excitation energies of 1.92 and 3.28 eV at 646 nm (HOMO → LUMO with 95% contribution) and 378 nm (H-1 → LUMO with 83% contribution) wavelengths, respectively. Compound **3** shows maximum absorbance at 488 nm (HOMO → LUMO with 98% contribution) and 341 nm (H-1 → LUMO with 98% contribution), where the excitation energies are 2.54 and 3.64 eV, respectively. In compound **4**, λ_abs_ values are at 682 nm (HOMO → LUMO with 96% contribution) and 388 nm (H-1 → LUMO with 89% contribution), where the excitation energies of these values are 1.82 and 3.19 eV, respectively. The computed UV–vis absorption spectra agree well with the experimental results.

**Table 2:** Maximum Absorbance Values of Electronic Transitions for Compounds **1–4**

solvent: the compound	experimental λ_abs_ (nm)	theoretical λ_abs_ (nm)	oscillator strength *f*	major contribution	excitation energy (eV)
**1**	500	473	0.77	HOMO → LUMO (98%)	2.62
	360	331	0.10	H-2 → LUMO (96%)	3.75
**2**	639	646	1.25	HOMO → LUMO (95%)	1.92
	367	378	1.98	H-1 → LUMO (83%)	3.28
**3**	512	488	0.78	HOMO → LUMO (98%)	2.54
	372	341	0.10	H-1 → LUMO (98%)	3.64
**4**	653	682	1.28	HOMO → LUMO (96%)	1.82
	374	388	1.64	H-1 → LUMO (89%)	3.19

**Figure 6:**
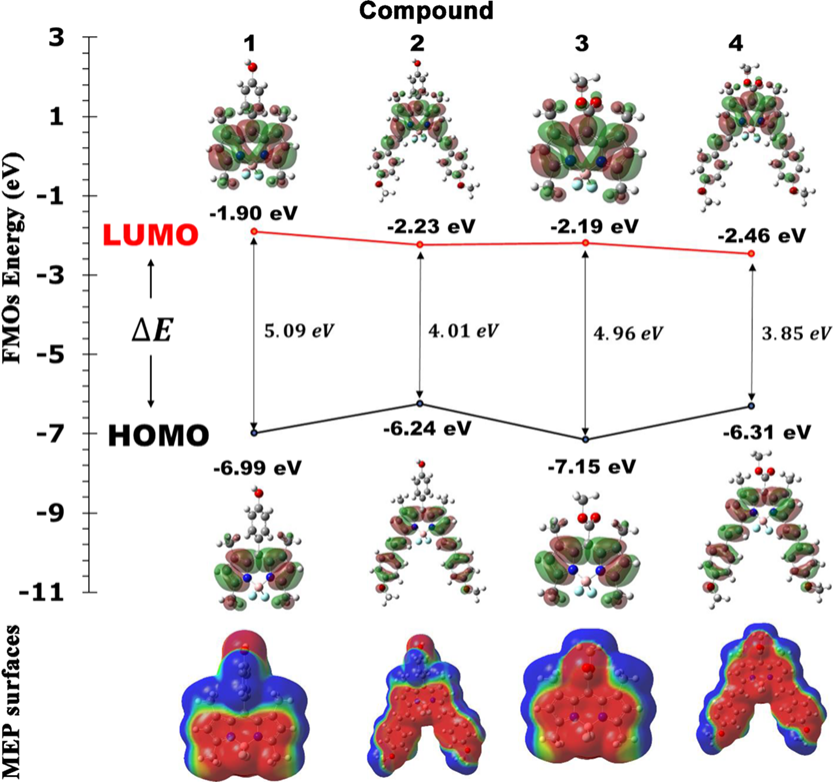
FMO Energy and MEP surfaces of compounds **1–4.**

To ascertain the chemical reactivity of the molecule and its ability to absorb light, an analysis of FMOs, also referred to as HOMOs and LUMOs, is performed. A molecule’s ability to be nucleophilic is determined by HOMO, while its electrophilic potential is determined by LUMO. The energy difference between these two orbitals (Δ*E*) helps in figuring out the stability. We performed calculations for *E*_HOMO_ and *E*_LUMO_, as well as the energy difference (Δ*E*) between HOMO and LUMO for the compounds listed in Table 2. In particular, compound **4** exhibited a smaller energy gap, which implies lower excitation energies for different excited states, increased chemical reactivity, increased reactivity (lower stability), and reduced chemical hardness (higher chemical softness). This suggests that compound **4** is more susceptible to photochemical activation. As shown in Figure 6, the isosurfaces of the HOMO/LUMO show red (positive charge) and green (negative charge) lobes, indicating delocalization of the charge density across the compound, while other regions show localization. The parameters such as ionization potential (IP), electron affinity (EA), hardness (η), electronegativity (χ), electrophilicity (ω), and softness (σ) were also calculated. The results are listed in Table 3. HOMO, indicating the electron acceptor areas, determines the ionization potential (IP = −*E*_HOMO_), while LUMO, which displays the electron acceptor areas, determines the electron affinity (EA = −*E*_LUMO_).^33^ Here, the calculated IPs were found to be 6.99, 6.24, 7.15, and 6.31 eV, and the values of EAs were found to be 1.90, 2.23, 2.19, and 2.46 for compounds **1**, **2**, **3,** and **4**, respectively. It is said that compound **4** exhibits a higher electron affinity (lower energy of the lowest unoccupied molecular orbital, *E*_LUMO_), indicating its enhanced capacity to readily accept electrons compared to other compounds.^34−36^ Additionally, we calculated the chemical hardness (, electronegativity (, and electrophilicity () indices using the HOMO and LUMO energy values. In fact, the compounds **1**, **2**, **3,** and **4** have chemical hardness values of 2.55, 2.01, 2.48, and 1.93 eV, electronegativity values of 4.45, 4.24, 4.67, and 4.39 eV, and electrophilicity values of 25.14, 17.98, 27.04, and 18.51 eV in the THF solvent, respectively. The results show that the electronegativity of compound **3** is higher than that of all of the other compounds, thus making it the best electron acceptor. According to the value of ω, compound **3** is also the strongest electrophile among all other compounds.^37,38^

**Table 3:** FMO Properties for Compounds **1–4** in THF Solvent

FMO properties | compound	1	2	3	4
*E*_HOMO_ (eV)	–6.99	–6.24	–7.15	–6.31
*E*_LUMO_ (eV)	–1.90	–2.23	–2.19	–2.46
Δ*E* **=** |*E*_HOMO_ **–** *E*_LUMO_| (eV)	5.09	4.01	4.96	3.85
ionization potential IP = −HOMO (eV)	6.99	6.24	7.15	6.31
electron affinity EA = *–*LUMO (eV)	1.90	2.23	2.19	2.46
hardness. (eV)	2.55	2.01	2.48	1.93
electronegativity. (eV)	4.45	4.24	4.67	4.39
electrophilicity. ω *=* χ^2^/2η (eV)	25.14	17.98	27.04	18.51
softness. σ = 1/ η (eV)	0.39	0.50	0.40	0.52

The determination of the MEP of a molecule is considered to be one of the most suitable approaches to identify sites within the molecule where intra- and intermolecular interactions occur. Figure 6 shows the MEP surfaces of the compounds studied. The red and yellow areas indicate the negative electrostatic potential, indicating electrophilic reactivity. Conversely, the blue region represents the positive electrostatic potential, associated with nucleophilic reactivity, while the green color denotes regions with zero potential. It is evident on the MEP surfaces of all compounds in Figure 6 that red areas in the BODIPY core have high electron densities, which indicates that electrophiles have electron-withdrawing reactive sites, while blue areas have a greater positivity, indicating that nucleophiles have electron-donating reactive sites. Now, let us focus on compounds **3** and **4** including −COOMe. The carbonyl carbon in a −COOMe ester functional group can exhibit a significant degree of polarization. This polarization arises from the difference in electronegativity between the C and O atoms of the carbonyl group (C=O). As a result, the oxygen atom tends to attract electron density toward itself, creating a partial negative charge on the oxygen atom and a partial positive charge on the carbon atom. Hence, it can be asserted that there is a charge transfer from the carbonyl carbon to the carbonyl oxygen (highlighted in red). The degree of polarization can vary depending on the specific ester and its substituents. For the −COOMe ester group, this substituent is an electron-donating methoxy group (−OCH_3_). In other words, the presence of an electron-donating group attached to the carbonyl carbon can change the positive charge density. Additionally, methyl group is also an electron-donating group, and it can be said that there is a charge transfer from the methyl group (indicated by the blue region) to the methoxy oxygen, as seen on the MEP surfaces in Figure 6.

An alternative method for assessing charge transfer during the electron excitation process is interfragment charge transfer (IFCT).^31^ In this study, we have carried out calculations to determine the charge transfer percentage (CT%) and its complement, the local excitation percentage (LE%), which are commonly used in electron excitation studies. These calculations focus specifically on the identified fragments in compounds **1, 2, 3**, and **4**, concerning the S_0_ → S_1_ transition. The results are very easy to understand from Table 4. First, the CT values increase in the order of compounds **4** > **2** > **3** = 1, while the LE values increase in the order of compounds **1** > **3** > **2** > **4**. For compounds **1** and **3,** the results show that during the S_0_ → S_1_ excitation, almost no net electron transfer occurred between fragments. Therefore, the values of LE (%) of compounds **1** and **3** are significantly larger than the CT (%), and these excitations can be mostly considered as local excitation states. On the other hand, it is worth emphasizing that the values of both the CT and LE states for compounds **2** and **4** seem to be very close. Typically, the ideal emissive state is achieved by combining both the local excitation (LE) and charge transfer (CT) state components, as this leads to a favorable combination of their individual advantages.^39^ In other words, the high efficiency of photoluminescence arises from the LE state, while the effective utilization of excitons is attributed to the CT state.

**Table 4:**
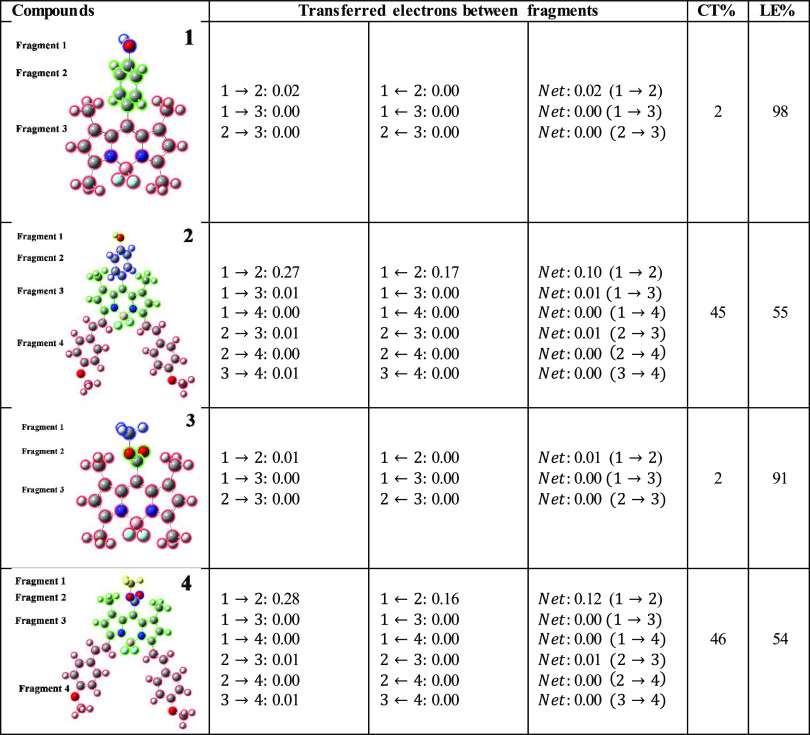
IFCT Analysis for Compounds **1–4** for the Electronic Transition S_0_ → S_1_ in the THF Solvent

## 4 Conclusions

A series of BODIPY dyes substituted by phenol or −COOMe units on the meso-position with and without a distyryl group, including methoxy units at the -C3 and -C5 positions of the BODIPY core, have been synthesized. All studied compounds demonstrate a strong absorption band in the UV–vis area. The synthesized dyes possessing π-conjugation with a methoxy moiety at the -C3 and -C5 positions of BODIPY lead to a bathochromic shift of about 120 nm. Furthermore, the fluorescence signals were significantly quenched by the attachment of a −COOMe unit to the meso-position of BODIPY due to the photoinduced electron transfer as well as intramolecular electron transfer. The performed OA Z-scan experiments found out that the BODIPY compounds with a distyryl group including a methoxy unit show a two-photon absorption character owing to the long conjugation length and, therefore, intramolecular electron transfer. Based on the OA Z-scan experiments, the TPA cross-section values were obtained as 74 and 81 GM for the compounds possessing phenol and −COOMe units treated by a distyryl group with methoxy moieties, respectively. Alternatively, it can be argued that the efficient utilization of excitation energy, arising from the combined effects of intercrossed excited states (LE and CT), is anticipated to enhance the overall efficiency. These findings were expected to contribute significantly to BODIPY research and offer valuable insights for improving the BODIPY chromophores with TPA properties in the near-infrared region, benefiting applications in bioimaging and photodynamic therapy processes.

## Supporting Information Available

^1^H NMR, ESI–MS, and decay kinetics of all the compounds (PDF).
