# Supplementary material for: Two-Photon Absorption Response of Functionalized BODIPY Dyes in Near-IR Region by Tuning Conjugation Length and Meso-Substituents
Source: ACS Omega. 2023 Aug 16;8(34):30939–48. doi: 10.1021/acsomega.3c02314 (PMC10468828; doi:10.1021/acsomega.3c02314)
Supplement: Supplementary file 1 — ao3c02314_si_001.pdf [file ao3c02314_si_001.pdf]

Supporting Information

Two-photon absorption response of functionalized BODIPY dyes in near IR region by tuning conjugation length and meso-substituents

Elif Akhuseyin Yildiz,[a,*] Bekir Asilcan Ünlü,[a] Ahmet Karatay,[a] Yasemin Bozkurt,[b] Muhammed Emre Özler,[c] Fazlı Sözmen,[c,*] Ebru Yabaş,[d,*] Bahadir Boyacioglu,[e] Hüseyin Ünver[f] and Ayhan Elmali[a]

[a] *Department of Physics Engineering, Faculty of Engineering, Ankara University, 06100 Beşevler-Ankara, Türkiye*

[b] *Sivas Cumhuriyet University, Department of Metallurgical and Materials Engineering, 58140, Sivas, Türkiye*

[c] *Sivas Cumhuriyet University, Nanotechnology Engineering Department, Faculty of Engineering, 58140, Sivas, Türkiye*

[d] *Sivas Cumhuriyet University, Advanced Technology Application and Research Center, 58140, Sivas, Türkiye*

[e] *Vocational School of Health Services, Ankara University, 06290 Kecioren-Ankara, Türkiye*

[f] *Department of Physics, Faculty of Science, Ankara University, 06100 Besevler-Ankara, Türkiye*

*Corresponding Author(s): eakhuseyin@ankara.edu.tr (Elif Akhuseyin Yildiz),
yabasebru@gmail.com (Ebru Yabaş),
fsozmen@cumhuriyet.edu.tr (Fazlı Sözmen)

ABSTRACT

BODIPY dyes substituted by phenol or -COOMe units at the meso-position (C8) with and without distyryl group including methoxy moiety at -C3,-C5 positions of the BODIPY have been synthesized to analyze photophysical properties. To clarify ground state interaction, absorption and emission features were investigated in the THF environment. Extending the π-conjugation with methoxy moiety at -C3,-C5 positions of the BODIPY leads to a spectral shifting of the absorption maxima towards red by 120 nm. In addition, attaching of -COOMe unit at meso-position of the BODIPY structure increase nonradiative molecular relaxation as compared to compounds possessing phenol substituent at the same position. We have investigated the effect of phenol and –COOMe group and π-extended conjugation length with methoxy moiety on the properties of two-photon absorption (TPA) and electron transfer dynamics by performing open aperture (OA) Z-scan and femtosecond transient absorption spectroscopy measurements, respectively. Synthesized BODIPY compounds with distyryl group including methoxy unit show TPA character due to longer conjugation length and therefore intramolecular charge transfer ability. Based on the OA Z-scan experiments upon photoexcitataion 800 nm pulsed laser light, TPA cross-section values were obtained as 74 GM and 81 GM for the compound possessing phenol and -COOMe unit at the meso position of BODIPY treated by distyryl group with methoxy moieties, respectively. Additionally, optical and electronic properties were calculated theoretically using the DFT method.

Keywords: Borondipyrromethene, Ultrafast pump-probe spectroscopy, Open aperture Z-scan technique, Two-photon absorption, DFT method

Figure S1. ^{1}H NMR of compound **1** 2
Figure S2. ESI - MS of compound **1** 3
Figure S3. ^{1}H NMR of compound **2** 3
Figure S4. ESI - MS of compound **2** 4
Figure S5. ^{1}H NMR of compound **3** 4
Figure S6. ESI - MS of compound **3** 4
Figure S7. ^{1}H NMR of compound 4 5
Figure S8. ESI - MS of compound **4** 5
Figure S9. Decay kinetics of compounds **a)** 2 and **b)** 4 by probing GSB and CTS wavelength in THF. 6
Figure S10. Decay traces of compounds **a) 2** and **b) 4** by probing GSB (around 650 nm) and ESA (around 465 nm) signal in THF. 6
Figure S11. Decay kinetics of compounds **a)** 2 and **b)** 4 by probing GSB in toluene, THF and ACN solutions. 7

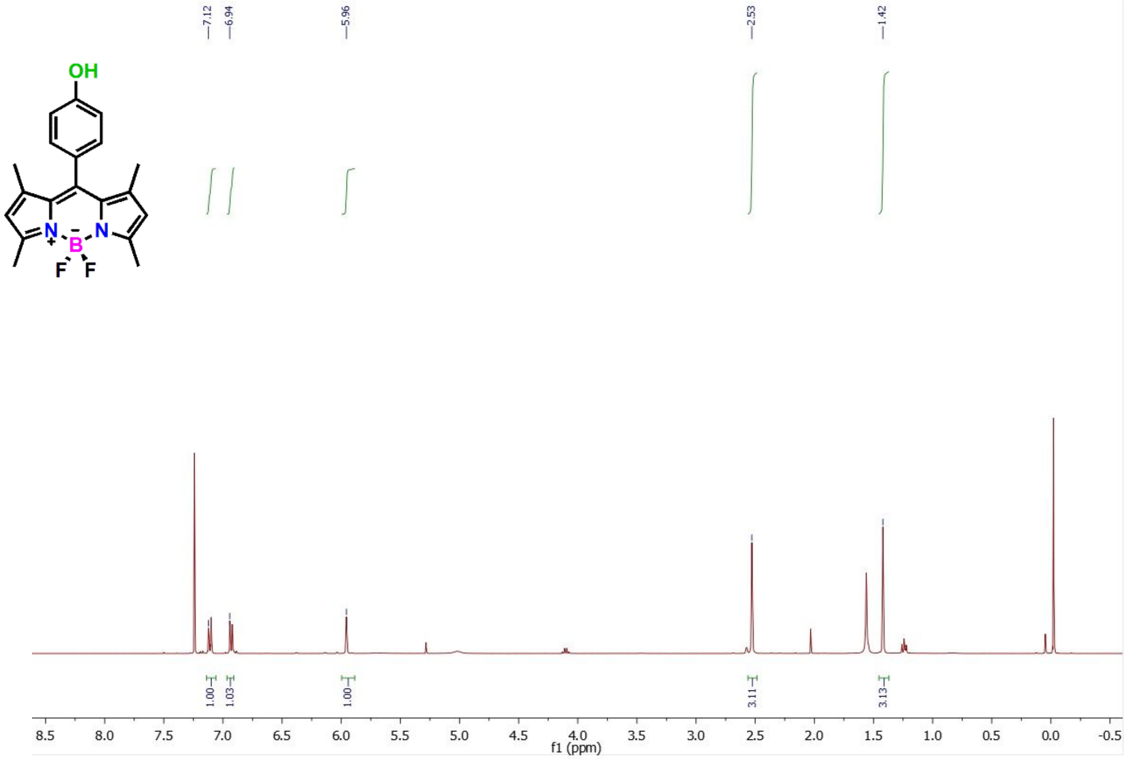

Figure S1. ^{1}H NMR of compound **1**

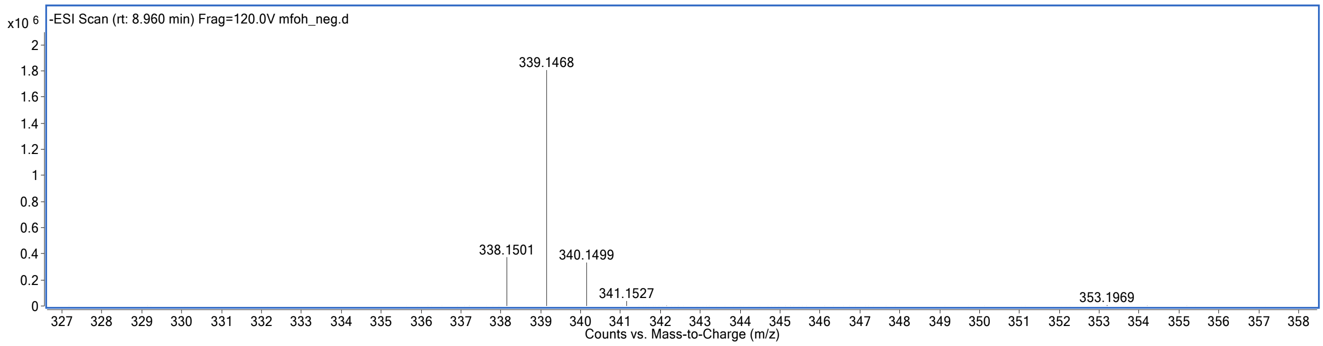

Figure S2. ESI - MS of compound **1**

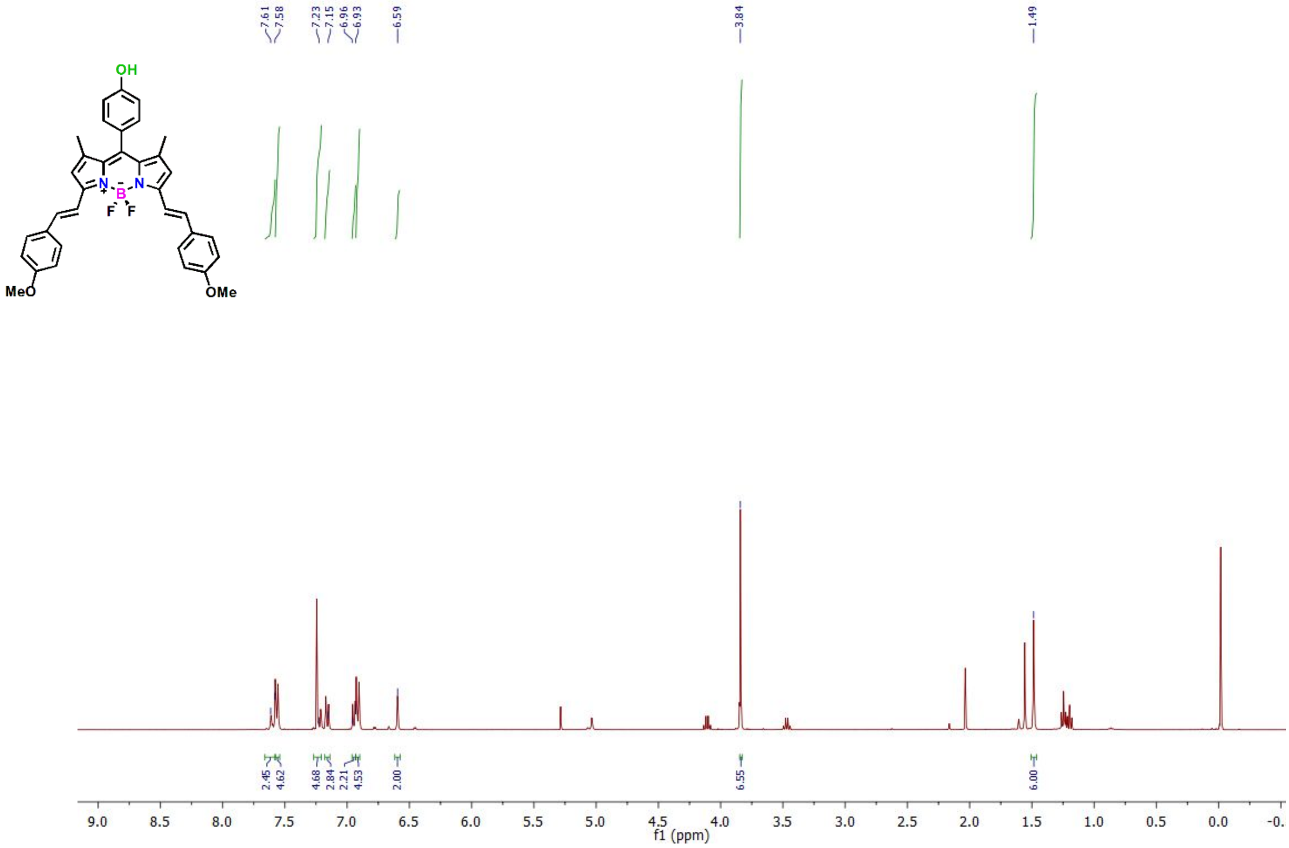

Figure S3. ^{1}H NMR of compound **2**

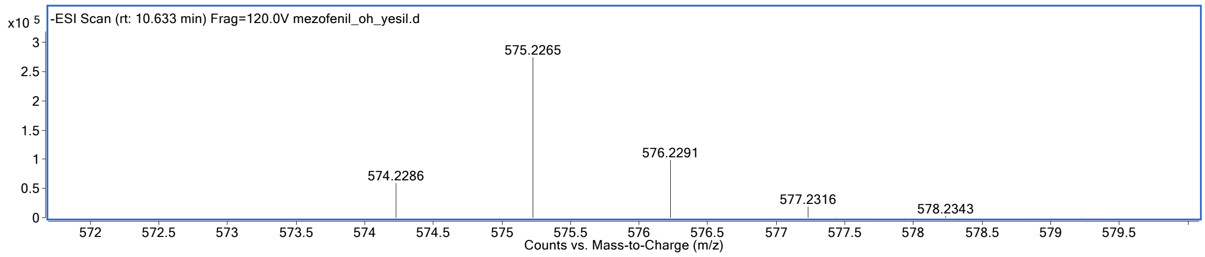

Figure S4. ESI - MS of compound **2**

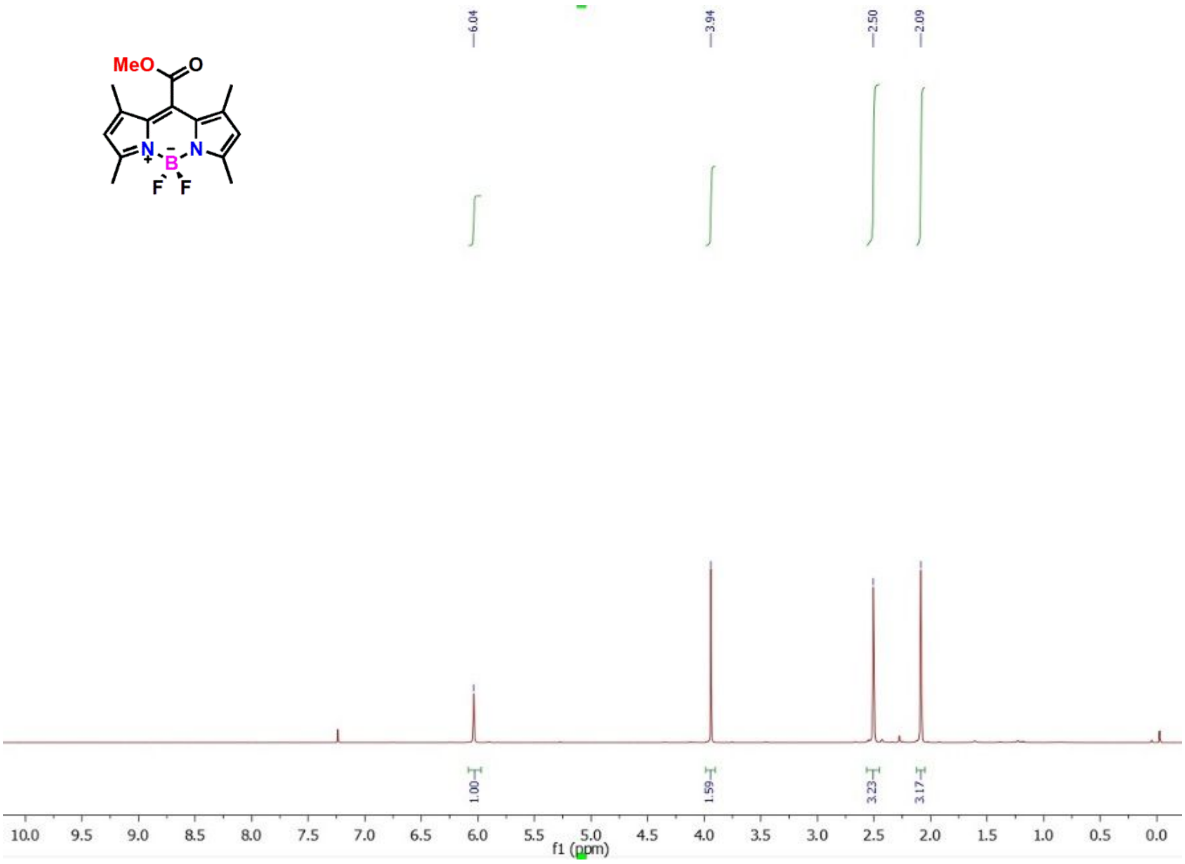

Figure S5. ^{1}H NMR of compound **3**

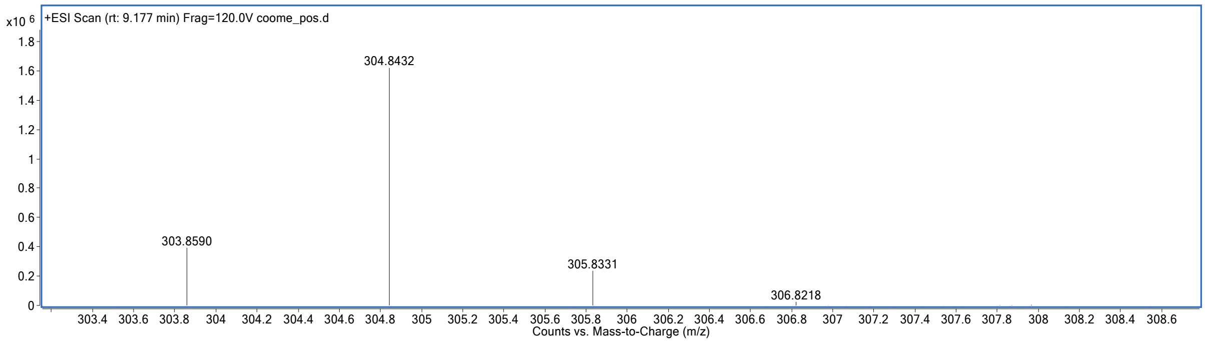

Figure S6. ESI - MS of compound **3**

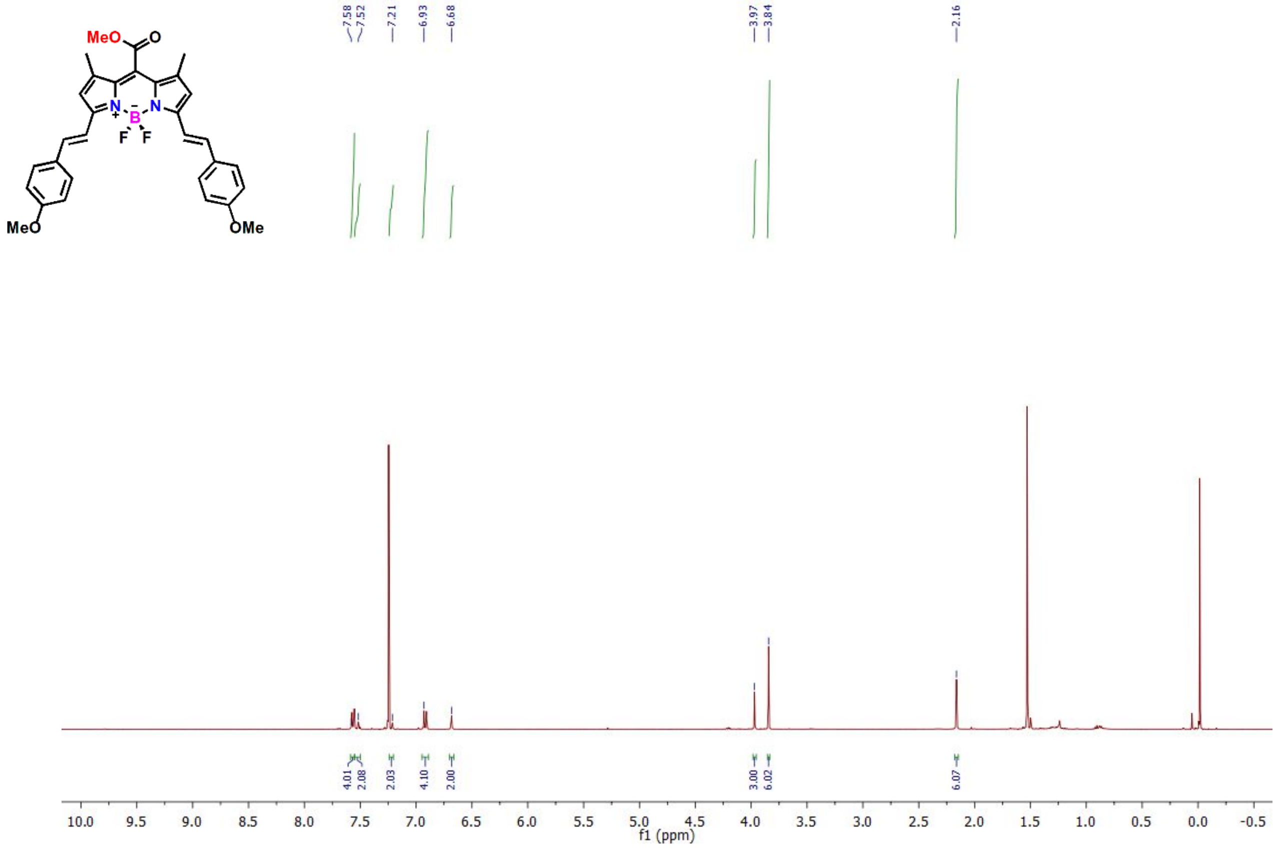

Figure S7. ^{1}H NMR of compound 4

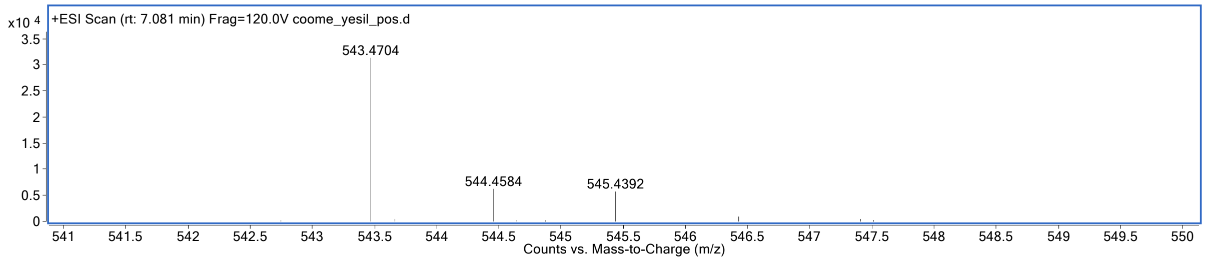

Figure S8. ESI - MS of compound **4**

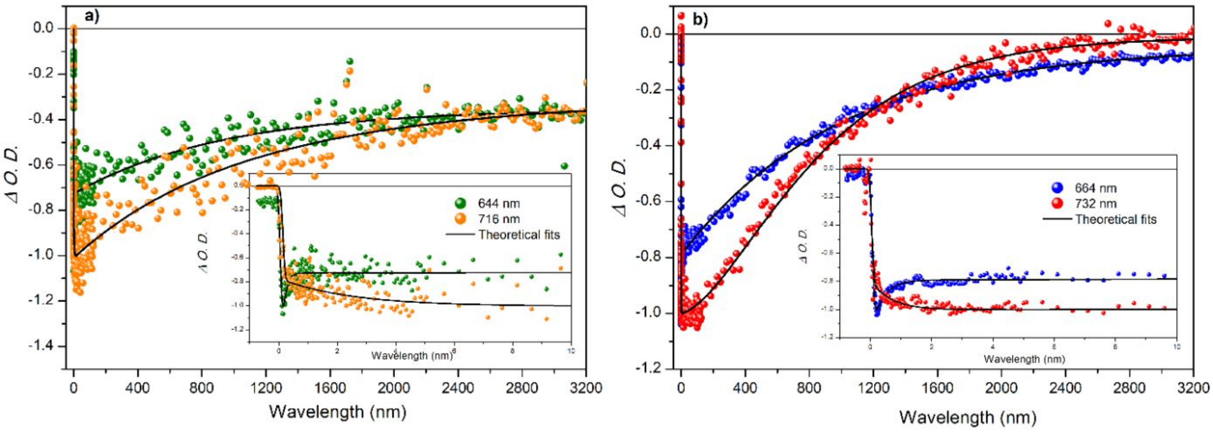

Figure S9. Decay kinetics of compounds **a) 2** and **b) 4** by probing GSB and CTS wavelength in THF.

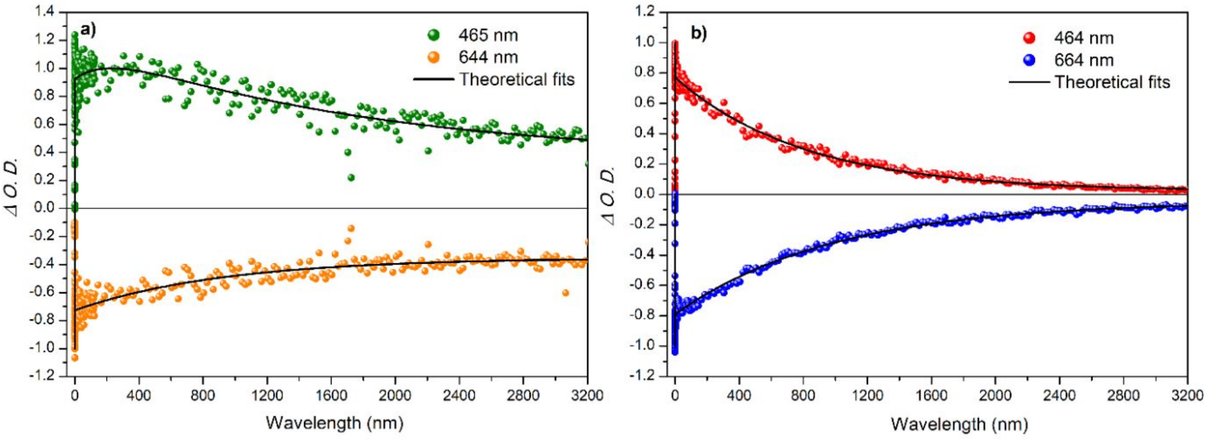

Figure S10. Decay traces of compounds **a) 2** and **b) 4** by probing GSB (around 650 nm) and ESA (around 465 nm) signal in THF.

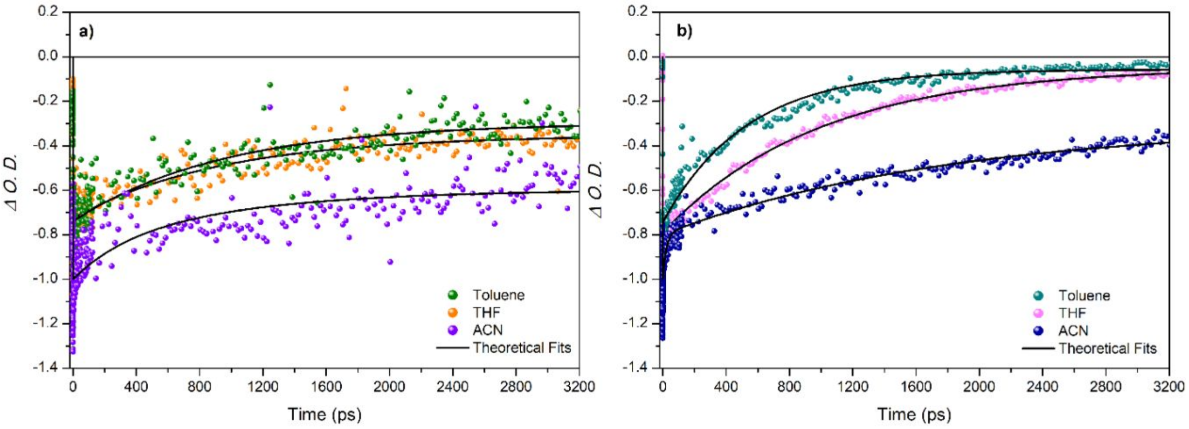

Figure S11. Decay kinetics of compounds **a)** 2 and **b)** 4 by probing GSB in toluene, THF and ACN solutions.
